# Alimentary Substances

**Published:** 1849-05

**Authors:** 


Of all the alimentary substances bread is one of the most nutritious, containing 80 per cent. of the materials of nutrition; beans and peas, however, contain as much as 92 to 93 per cent. Butcher's meat contains 35;—potatoes 25, carrots 14, greens and turnips 8 per cent. Thus a pound of good bread is equal to from two pounds and a half to three pounds of the best potatoes, and 75 pounds of bread with 30 pounds of meat equal 300 pounds of potatoes; and what is more remarkable a pound of rice or beans is equal to three pounds of potatoes.

